# PTEN status, tumor immune microenvironment, and survival in colorectal cancer

**DOI:** 10.1007/s00428-025-04327-8

**Published:** 2025-11-18

**Authors:** Joni Karjalainen, Onni Sirkiä, Päivi Sirniö, Hanna Elomaa, Henna Karjalainen, Ville K. Äijälä, Meeri Kastinen, Vilja V. Tapiainen, Vesa-Matti Pohjanen, Maarit Ahtiainen, Olli Helminen, Erkki-Ville Wirta, Taneli T. Mattila, Outi Lindgren, Jukka Rintala, Sanna Meriläinen, Juha Saarnio, Tero Rautio, Toni T. Seppälä, Jan Böhm, Jukka-Pekka Mecklin, Anne Tuomisto, Markus J. Mäkinen, Juha P. Väyrynen

**Affiliations:** 1 Translational Medicine Research Unit, Medical Research Center Oulu, Oulu University Hospital, and University of Oulu, Aapistie 5A, 90220 Oulu, Finland; 2 Department of Environmental and Biological Sciences, University of Eastern Finland, Kuopio, Finland; 3 Research Program in Systems Oncology, University of Helsinki, Helsinki, Finland; 4 Central Finland Biobank, Well Being Services County of Central Finland, Hospital Nova of Central Finland, Jyväskylä, Finland; 5 Department of Gastroenterology and Alimentary Tract Surgery, Tampere University Hospital, Tampere, Finland; 6 Faculty of Medicine and Health Technology, Tampere University and Tays Cancer Centre, Tampere University Hospital, Tampere, Finland; 7 Department of Gastrointestinal Surgery, Helsinki University Central Hospital, University of Helsinki, Helsinki, Finland; 8 Applied Tumour Genomics, Research Program Unit, University of Helsinki, Helsinki, Finland; 9 Department of Pathology, Well Being Services County of Central Finland, Hospital Nova of Central Finland, Jyväskylä, Finland; 10 Faculty of Sport and Health Sciences, University of Jyväskylä, Jyväskylä, Finland; 11 Department of Education and Research, Well Being Services County of Central Finland, Hospital Nova of Central Finland, Jyväskylä, Finland

**Keywords:** PTEN protein, Tumor immune microenvironment, Immunohistochemistry, Colorectal cancer, Prognostic marker

## Abstract

Phosphatase and tensin homolog (PTEN) is a tumor suppressor involved in cell proliferation, DNA repair, apoptosis, and cell cycle regulation. Its loss has been linked to worse prognosis and poor immune therapy response in several cancers, but findings in colorectal cancer (CRC) have been inconsistent. This study aims to evaluate the prognostic value of PTEN expression and its relationship with the tumor immune microenvironment in two large CRC cohorts (combined *N* = 2303). PTEN expression was assessed by immunohistochemistry and categorized as intact, reduced, or lost. Additionally, three multiplex immunohistochemistry assays were used to assess immune cell composition and expression of immunosuppressive markers within the tumor environment. PTEN loss was observed in 12% of tumors in cohort 1 and 11% in cohort 2. PTEN expression status showed no significant prognostic value. For CRC-specific mortality, the multivariable HR for PTEN loss (vs. intact expression) was 1.19 (95% CI 0.88–1.61) in cohort 1 and 0.85 (95% CI 0.55–1.31) in cohort 2. PTEN loss was associated with *BRAF* mutations and mismatch repair (MMR) deficiency in both cohorts, but was not independently associated with tumor immune cell composition or expression of PD-L1, PD-1, IDO, and ARG1. In conclusion, PTEN immunohistochemistry lacked prognostic value in CRC and did not reflect the tumor immune landscape. These findings suggest that PTEN immunohistochemistry alone may have limited clinical utility as a biomarker in CRC, highlighting the need for complementary genomic profiling in future studies.

**Supplementary Information:**

The online version contains supplementary material available at 10.1007/s00428-025-04327-8.

## Introduction

Colorectal cancer (CRC) is the third most prevalent cancer worldwide, and second leading cause of cancer related mortality with nearly a million deaths annually [1]. CRC incidence rates are increasing faster than those of other common cancer types [2]. The rising incidence is a global health concern, and there is a need for better prognostic factors and screening methods. Among the molecular pathways that are deregulated in CRC, the PI3K/AKT signaling cascade plays a crucial role in regulating cell growth, survival, metabolism, and invasion [3, 4].

Phosphatase and tensin homolog (PTEN) is a dual-specificity phosphatase that dephosphorylates both lipids and proteins. Its main function is to antagonize the PI3K/AKT pathway reducing the activation of AKT [5]. PTEN also has protein phosphatase activity targeting multiple substrates involved in cell cycle regulation, DNA repair, apoptosis, and cytoskeleton dynamics [6, 7]. Inactivating mutations of *PTEN* are recognized in several cancers, including glioblastoma, lung cancer, endometrial cancer, breast cancer, and prostate cancer [3, 6]. In CRC, around 8–12% of cases harbor *PTEN* loss due to point mutations and/or deletion. Additionally, PTEN expression can be down-regulated by methylation, with some studies reporting PTEN loss prevalence as high as 30–40%, depending on methodological differences and study populations [8–11]. The prognostic value of PTEN expression in CRC patients has remained ambiguous, primarily due to limitations in sample sizes and inconsistencies in survival endpoint reporting, including progression free survival versus overall survival [12].

More recent studies have unveiled that PTEN loss may facilitate immune evasion through multiple mechanisms, such as upregulating programmed death-ligand 1 (PD-L1) expression on tumor cells, thereby suppressing the T cell anti-tumor response [13]. Furthermore, PTEN loss has been associated with increased infiltration of immunosuppressive cell populations within the tumor microenvironment (TME), including M2 macrophages, regulatory T cells (Treg), and myeloid-derived suppressor cells (MDSC) [13].

Loss of PTEN expression in CRC has largely been evaluated in smaller studies with differing assessment methods. This study aims to investigate the prognostic significance of PTEN protein expression in two large CRC cohorts (*N* = 2303) and analyze how PTEN expression status impacts the tumor immune microenvironment.

## Methods

### Study population

The study examined two distinct CRC cohorts (Fig. S1A). Cohort 1 (*N* = 1343) was retrospectively assembled from patients treated at Central Finland Central Hospital between the years 2000 and 2015 [14]. The second cohort, cohort 2 (*N* = 1011) was prospectively collected between 2006 and 2020 from patients treated at Oulu University Hospital [15].

Both cohorts included patients who had undergone surgical resection with available tumor specimens. Cases with unsuccessful PTEN immunohistochemistry were excluded (cohort 1, *N* = 16; cohort 2, *N* = 35). To mitigate confounding effects of surgical complications, patients who died within 30 days of surgery were excluded from CRC-specific and overall survival assessment (cohort 1, *N* = 38; cohort 2, *N* = 5).

For the immune cell analyses in cohort 1, patients who had received preoperative radiotherapy or combined chemoradiotherapy were excluded (*N* = 238) to avoid potential confounding effects on the immune microenvironment [16]. Cases with missing immune cell data were additionally excluded (*N* = 25–48 depending on the immune cell panel).

### Histopathological analyses

Primary tumor resection samples were fixed in 10% formalin and embedded in paraffin. Using hematoxylin and eosin (H&E) stained sections, the pathological characteristics of the tumors had been previously evaluated, including TNM-stage, grade, and lymphovascular invasion status [14, 17, 18]. TNM staging was performed according to the criteria established by the Union for International Cancer Control (UICC) [19]. Grading of the tumors was conducted according to the WHO criteria. Additionally, previous studies had analyzed mismatch repair (MMR) status, TP53 expression pattern and *BRAF* V600E mutation specific immunohistochemistry within these CRC cohorts [14, 17, 18, 20].

### PTEN immunohistochemistry

PTEN status was evaluated using an immunohistochemistry assay from tissue microarrays (TMAs) that were designed to contain four 1-mm diameter cores per tumor. The immunohistochemistry assay was performed on Leica Bond III immunohistochemistry stainer, using D4.3 antibody clone (Cell Signaling Technology, 9188S, dilution 1:100, incubation 30 min), Leica Bond ER2 (Leica AR9640) pretreatment, and Leica Bond Polymer Refine Detection kit (Leica DS9800).

**Fig. 1:**
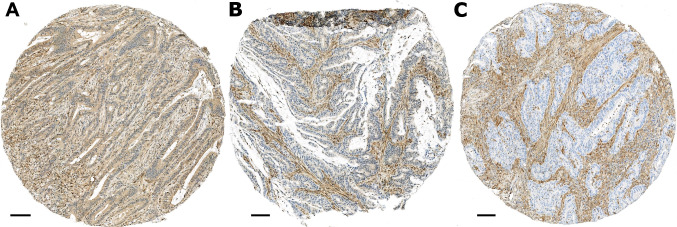
Representative examples of the three PTEN expression categories. **A** A tissue microarray (TMA) core showing fully intact PTEN expression. Uniform, strong nuclear and cytoplasmic staining both in the stroma and tumor cells. **B** An example core of a reduced PTEN expression tumor. Here, the tumor cells have some positivity in the nuclei and faint, but visible cytoplasmic staining compared to the surrounding stromal tissue. **C** Complete loss of PTEN expression with no nuclear or cytoplasmic staining of the tumor cells. Scalebar size is 100 µm

PTEN expression was classified into three groups: total loss of expression, reduced expression, and intact expression (Fig. 1). Lost expression was determined in cases with sufficient stromal staining (internal positive control), including nuclear positivity. Reduced expression was defined as significantly weaker cytoplasmic and nuclear staining in tumor cells compared to stromal cells, but not entirely absent. Intact expression was classified when tumor cells exhibited strong positive nuclear staining that was comparable to or stronger than the staining observed in stromal cells.

To assess the concordance between TMA-based and whole-slide PTEN expression classification, we performed validation analysis using 30 samples from cohort 2. PTEN expression was scored into three groups using the same system as in the main analysis. Agreement between whole-slide and TMA-based assessments was evaluated using Cohen’s κ statistic.

### Immune cell analysis

Tumor immune cell densities for both the tumor epithelial and stromal compartments have been previously assessed with digital image analysis of multiplex immunohistochemistry [14, 21–23] (Fig. 3). The recognized cell types included CD3^+^ T cells, CD20^+^CD79A^+^ B cells, CD20^–^CD79A^+^ plasma cells, M1-like and M2-like macrophages, CD14^+^HLA-DR^+^ mature monocytic cells, CD14^+^HLA-DR^-^ immature monocytic cells, CD66B^+^ granulocytes, and tryptase^+^ mast cells. Macrophage phenotyping was based on a polarization index of four separate markers [21]. The same assays were used to evaluate the densities of four potentially immunosuppressive cell populations (PD-L1^+^ macrophages, PD-1^+^ T cells, IDO^+^ monocytic cells, ARG1^+^ granulocytic cells). In addition, PD-L1 and IDO expression levels were evaluated in tumor cells using the histoscore system. Histoscore was defined as the product of staining intensity (on a 0–3 scale) and the percentage of positive tumor cells, resulting in a score ranging from 0 to 300.

### Statistical analysis

Statistical analyses were executed with R studio (version 2024.09.1; R version 4.4.1) using the following libraries: *scales* (1.3.0), *survival* (3.6–4), *survminer* (0.5.0), *ggpubr* (0.6.0), *haven* (2.5.4), *gmodels* (2.19.1), *tidyverse* (2.0.0), *conflicted* (1.2.0). P values less than 0.05 were deemed statistically significant. The three-category PTEN variable (intact, reduced, lost) was used in all analyses.

Crosstabulation and the chi-square test were used to evaluate the associations between PTEN expression loss and various tumor and patient characteristics. Associations with immune cell densities were evaluated using the Kruskal–Wallis test. Cox regression models and Kaplan–Meier curves were used for evaluating both CRC-specific and overall survival. The proportional hazards assumption for Cox regression models was checked using Schoenfeld residuals. In the multivariate models, age, sex, tumor stage, tumor budding, lymphovascular invasion, grade, year of operation, tumor location, preoperative radiotherapy/chemotherapy, *BRAF* status, and MMR status were included as covariates.

## Results

### PTEN loss is associated with mismatch repair deficiency and *BRAF* mutation

The study included two independent CRC cohorts (*N* = 1327 and *N* = 976). Key clinicopathologic characteristics were evaluated and compared based on PTEN expression status (Table 1). The prevalence of PTEN loss was comparable between cohort 1 (12%) and cohort 2 (11%). Significant associations were found for *BRAF* and MMR-status, where PTEN loss was more common in *BRAF*-mutated tumors and those with MMR deficiency. Additionally, proximal tumor location was more frequent with loss of PTEN expression across both cohorts. PTEN expression category did not show statistically significant relationships with TNM stage, tumor grade, or lymphovascular invasion. Reduced PTEN expression was associated with TP53 mutant expression pattern in cohort 2 (*p* = 0.0066) but not significantly in cohort 1 (*p* = 0.18).

**Table 1:** Patient and tumor characteristics and their associations with PTEN expression in cohorts 1 and 2

Characteristic	Cohort 1					Cohort 2				
	Total *N*	PTEN			*p* value	Total N	PTEN			*p* value
		Intact	Reduced	Lost			Intact	Reduced	Lost	
All cases	1327 (100%)	950 (72%)	212 (16%)	165 (12%)		976 (100%)	749 (77%)	124 (13%)	103 (11%)	
Sex					0.10					0.012
Female	619 (47%)	452 (48%)	85 (40%)	82 (50%)		426 (44%)	329 (44%)	42 (34%)	55 (53%)	
Male	708 (53%)	498 (52%)	127 (60%)	83 (50%)		550 (56%)	420 (56%)	82 (66%)	48 (47%)	
Age (years)					0.77					0.77
< 65	396 (30%)	275 (29%)	67 (32%)	54 (33%)		331 (34%)	252 (34%)	44 (36%)	35 (34%)	
65–75	451 (34%)	323 (34%)	74 (35%)	54 (33%)		357 (37%)	277 (37%)	47 (38%)	33 (37%)	
> 75	480 (36%)	352 (37%)	71 (34%)	57 (36%)		288 (30%)	220 (29%)	33 (27%)	35 (30%)	
Tumor location					0.00053					0.00010
Proximal colon	530 (40%)	387 (41%)	60 (28%)	83 (50%)		321 (33%)	249 (33%)	27 (22%)	45 (44%)	
Distal colon	401 (30%)	286 (30%)	76 (36%)	39 (24%)		206 (21%)	172 (23%)	18 (15%)	16 (16%)	
Rectum	396 (30%)	277 (29%)	76 (36%)	43 (26%)		449 (46%)	328 (44%)	79 (64%)	42 (41%)	
Neoadjuvant treatment					0.44					0.0025
No	1089 (82%)	777 (82%)	171 (81%)	141 (86%)		760 (78%)	593 (79%)	82 (66%)	85 (82%)	
Yes	238 (18%)	173 (18%)	41 (19%)	24 (15%)		216 (22%)	156 (21%)	42 (34%)	18 (18%)	
Disease stage					0.26					0.31
I	245 (19%)	169 (18%)	44 (21%)	32 (19%)		213 (22%)	168 (22%)	25 (20%)	20 (19%)	
II	485 (37%)	363 (38%)	66 (31%)	56 (34%)		308 (32%)	241 (32%)	38 (31%)	29 (28%)	
III	425 (32%)	292 (31%)	80 (38%)	53 (32%)		346 (36%)	257 (34%)	52 (42%)	37 (36%)	
IV	172 (13%)	126 (13%)	22 (10%)	24 (15%)		109 (11%)	83 (11%)	9 (7.3%)	17 (17%)	
Grade					0.20					0.27
Low grade	1106 (83%)	795 (84%)	181 (85%)	130 (79%)		832 (85%)	646 (86%)	102 (82%)	84 (82%)	
High grade	221 (17%)	155 (16%)	31 (15%)	35 (21%)		144 (15%)	103 (14%)	22 (18%)	19 (18%)	
Lymphovascular invasion					0.80					0.080
No	1040 (78%)	747 (79%)	167 (79%)	126 (76%)		512 (53%)	407 (54%)	60 (48%)	45 (44%)	
Yes	287 (22%)	203 (21%)	45 (21%)	39 (24%)		464 (48%)	342 (46%)	64 (52%)	58 (56%)	
Tumor budding					0.41					0.00011
Grade 1 (0–4)	971 (73%)	695 (73%)	151 (71%)	125 (76%)		642 (66%)	513 (69%)	69 (56%)	60 (58%)	
Grade 2 (5–9)	211 (16%	158 (17%)	31 (15%)	22 (13%)		181 (19%)	128 (17%)	22 (18%)	31 (30%)	
Grade 3 (≥ 10)	145 (11%)	97 (10%)	30 (14%)	18 (11%)		153 (16%)	108 (14%)	33 (27%)	12 (12%)	
MMR status					0.0077					0.0021
Proficient	1158 (87%)	825 (87%)	197 (93%)	136 (82%)		849 (87%)	649 (87%)	118 (95%)	82 (80%)	
Deficient	169 (13%)	125 (13%)	15 (7.1%)	29 (18%)		127 (13%)	100 (13%)	6 (4.8%)	21 (20%)	
*BRAF* status^A^					0.0017					0.00076
Wild type	1139 (86%)	817 (86%)	193 (91%)	129 (78%)		865 (89%)	667 (89%)	117 (94%)	81 (79%)	
Mutant	187 (14%)	132 (14%)	19 (9.0%)	36 (22%)		111 (11%)	82 (11%)	7 (5.6%)	22 (21%)	
TP53 expression pattern					0.18					0.0066
Wild type	580 (44%)	419 (44%)	82 (39%)	79 (48%)		374 (38%)	297 (40%)	32 (26%)	45 (44%)	
Mutant	747 (56%)	531 (56%)	130 (61%)	86 (52%)		602 (62%)	452 (60%)	92 (74%)	58 (56%)	

*MMR* mismatch repair

A: Data missing for one case in cohort 1

Whole-slide validation of TMA PTEN immunohistochemistry was performed on 30 cases from cohort 2. Of these, 28 (93%) were identically classified, while two cases differed between “intact” and “reduced” expression categories. Agreement between TMA-based and whole-slide assessments was substantial (Cohen’s *κ* = 0.80).

### PTEN status does not predict survival

**Fig. 2:**
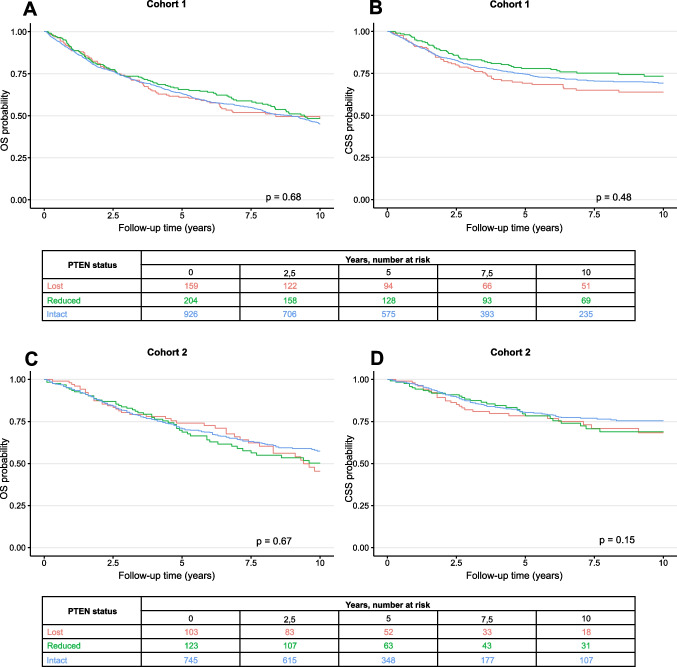
Kaplan–Meier survival analyses according to PTEN expression categories. **A**, **B** Survival analysis graphs for cohort 1, overall survival (OS) (**A**) and cancer-specific survival (CSS) (**B**) shown separately. **C**, **D** Corresponding survival curves for Cohort 2. PTEN expression categories are consistently color-coded across all panels: red for lost expression, green for reduced expression, and blue for intact expression. Abbreviations: CSS, cancer-specific survival; OS, overall survival

During a 10-year follow-up period, there were 646 deaths, including 354 CRC-related deaths in cohort 1 and 331 deaths, including 198 CRC-related deaths in cohort 2. The median follow-up time for censored cases was 10.0 years (IQR 7.3–10.0) in cohort 1 and 6.0 years (IQR 4.0–9.9) in cohort 2. Cox regression models revealed no statistically significant associations between PTEN expression status and patient survival outcomes, including overall survival and cancer-specific survival (Table 2). For cancer-specific survival, the multivariable hazard ratio (HR) for PTEN loss (compared to intact PTEN expression) was 1.19 (95% CI 0.88–1.61) in cohort 1 and 0.85 (0.55–1.31) in cohort 2. The Kaplan–Meier curves (Fig. 2) further supported these findings, showing that PTEN loss did not significantly impact patient survival.

**Table 2:** Univariable and multivariable Cox regression models for cancer-specific survival and overall survival according to PTEN expression status

	No. of cases	Colorectal cancer-specific survival			Overall survival		
		No. of events	Univariable HR (95% CI)	Multivariable HR (95% CI)	No. of events	Univariable HR (95% CI)	Multivariable HR (95% CI)
Cohort 1							
PTEN status							
Intact	926	254	1 (referent)	1 (referent)	469	1 (referent)	1 (referent)
Reduced	204	47	0.81 (0.60–1.12)	0.83 (0.61–1.15)	98	0.91 (0.73–1.13)	0.95 (0.76–1.19)
Lost	159	53	1.21 (0.90–1.62)	1.19 (0.88–1.61)	79	0.96 (0.76–1.22)	0.97 (0.76–1.24)
*P*_*trend*_			0.54	0.57		0.55	0.72
Cohort 2							
PTEN status							
Intact	745	143	1 (referent)	1 (referent)	242	1 (referent)	1 (referent)
Reduced	123	29	1.16 (0.78–1.73)	1.06 (0.69–1.64)	49	1.16 (0.82–1.52)	1.09 (0.79–1.51)
Lost	103	26	1.26 (0.83–1.91)	0.85 (0.55–1.31)	40	1.11 (0.80–1.55)	0.80 (0.57–1.13)
*P*_*trend*_			0.23	0.55		0.42	0.33

*CI* confidence interval, *HR* hazard ratio

Multivariable Cox proportional hazards regression models were adjusted for sex, age (< 65, 65–75, > 75), year of operation (2005–2010, 2011–2015, 2016–2020), tumor location (proximal colon, distal colon, rectum), preoperative radiotherapy/chemoradiotherapy (no, yes), disease stage (I–II, III, IV), tumor grade (well/moderately differentiated, poorly differentiated), lymphovascular invasion (negative, positive), mismatch repair (MMR) status (proficient, deficient), *BRAF* status (wild type, mutant)

### PTEN status is not associated with the composition of the tumor immune microenvironment

**Fig. 3:**
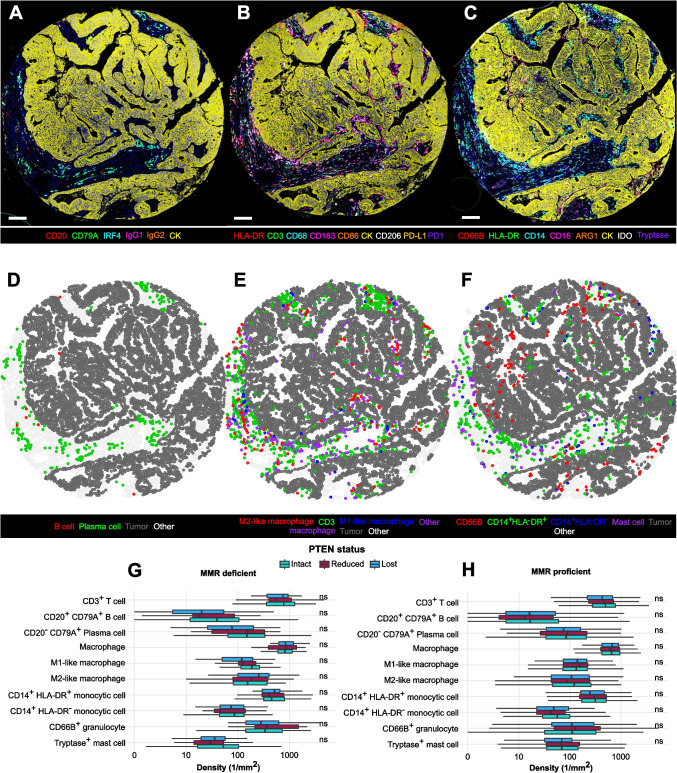
Representative example images of the multiplex-immunohistochemistry assays used for immune cell density analysis and boxplots of immune cell densities in tumor samples based on PTEN expression status. **A**, **B**, **C** Examples of multiplex immunohistochemistry images of colorectal carcinoma. **D**, **E**, **F** Matching immune cell maps created using machine learning-assisted analysis. The assays were performed for cohort 1: CD3^+^ T cells, macrophages, M1-like macrophages, and M2-like macrophages (*N* = 1060); CD14^+^HLA-DR^+^ mature monocytic cells, CD14^+^HLA-DR- immature monocytic cells, CD66B^+^ granulocytes, and tryptase^+^ mast cells (*N* = 1041); CD20^+^CD79A^+^ B cells and CD20^–^ CD79A^+^ plasma cells (*N* = 1064). Scalebar indicates 100 µm. **G**, **H** Immune cell densities across PTEN expression categories for MMR-deficient and MMR-proficient tumors. PTEN categorization shown at the top, intact expression plotted with green, reduced with red and lost expression with blue. Abbreviations: ns, not significant (Kruskal–Wallis test *p* value > 0.05); MMR, mismatch repair

The immune cell microenvironment was analyzed in cohort 1 using three multiplex immunohistochemistry panels (Fig. 3). Immune cell densities did not significantly differ across PTEN expression categories. This finding held true for both MMR deficient and proficient tumors, which were separately evaluated, considering the association of PTEN loss with MMR deficiency.

Four potentially immunosuppressive cell populations (PD-L1^+^ macrophages, PD-1^+^ T cells, IDO^+^ monocytic cells, ARG1^+^ granulocytic cells) were also evaluated using the same multiplex assays (Fig. S2), but no significant differences were observed across PTEN expression groups in MMR-deficient or MMR-proficient tumors. Similarly, PD-L1 and IDO expression in tumor cells was not associated with PTEN expression status.

## Discussion

This study aimed to evaluate the prognostic significance of PTEN expression in CRC and its impact on the tumor immune microenvironment. Despite the comprehensive analysis of two large cohorts, our findings indicate that PTEN expression status, as determined by immunohistochemistry, does not significantly impact patient survival outcomes or immune cell densities within the tumor microenvironment.

Our results contradict with several studies that have reported correlations between PTEN loss and reduced survival time in CRC patients [8, 11, 12, 24]. However, our analysis was limited to immunohistochemical assessment of PTEN expression, which may not fully capture the biological complexity underlying PTEN inactivation. The mechanisms of PTEN loss are heterogeneous and include genetic mutations, deletions, and epigenetic modifications, each of which may have distinct prognostic implications. In support of this notion, a recent genomic study found that *PTEN* deletions, but not other types of mutations, were associated with worse prognosis [25]. That study also highlighted the modifying effect of microsatellite instability with particularly poor outcomes observed in microsatellite stable (MMS) tumors harboring *PTEN* deletions.

Furthermore, emerging evidence indicates that the prognostic impact may differ between heterozygous and homozygous *PTEN* deletions, with heterozygous loss being more common and associated with genomic instability, immune evasion, and poor outcomes across multiple cancer types including CRC [26]. Although immunohistochemistry offers the advantage of detecting protein loss regardless of the underlying cause, it cannot resolve these genomic distinctions. In our study, we attempted to address this in part by classifying PTEN expression into three categories, also capturing the intermediate expression cases. Future studies integrating immunohistochemistry with genomic profiling are warranted to identify subsets of CRC cases where immunohistochemical PTEN loss might be prognostically significant.

An important consideration in this study is the use of TMAs for PTEN immunohistochemistry. While four 1-mm cores per tumor were sampled to minimize sampling variability, this approach may not fully capture intratumoral heterogeneity, particularly given the documented association of *PTEN* alterations with genomic instability. Consequently, tumors with focal PTEN loss may have been misclassified, potentially attenuating observed associations with clinical outcomes*.* Consistent with this, our whole-slide validation showed high but incomplete concordance with TMA-based scoring (Cohen’s *κ* = 0.80).

We found significant associations between PTEN loss and both MMR deficiency and *BRAF* mutations. This aligns with previous research indicating that PTEN loss is more often observed in microsatellite unstable tumors [25]. *BRAF* mutations have also been found to co-occur with *PTEN* mutations especially in microsatellite stable tumors [25]. These findings suggest that PTEN loss is more common in tumors developing through the serrated pathway of colorectal carcinogenesis [27]. These tumors comprise around 10 to 30% of all CRCs and are characterized by their specific precursor lesions such as sessile serrated lesion that both morphologically and molecularly differ from conventional adenomas [27]. Despite these findings, PTEN expression was not independently associated with invasive CRC characteristics or survival outcomes in our study.

The analysis of immune cell densities revealed no significant differences between tumors with intact, reduced, or lost PTEN expression. Our study utilized three custom multiplex immunohistochemistry panels, allowing for more detailed immune cell phenotyping compared to conventional single-color immunohistochemistry. For instance, macrophage phenotypes (M1-like and M2-like) were identified using a combination of four polarization markers, as there is no single specific marker for these phenotypes [28]. Previous reports have suggested that PTEN loss can facilitate immune evasion by upregulating PD-L1 expression and by increasing the immunosuppressive cell infiltration in the tumor environment [13, 29]. However, in our analysis, PTEN loss was not associated with changes in the densities of any immune cell types, including the evaluated immunosuppressive cell populations. Furthermore, no significant upregulation of PD-L1 or IDO expression was observed in tumor cells across PTEN expression groups. These findings suggest that PTEN loss does not consistently influence the tumor immune microenvironment in CRC. It is possible that the impact of PTEN on immune modulation may depend on the specific underlying mechanism of its inactivation.

The authors acknowledge that a limitation of this work was the lack of detailed genetic profiling to differentiate the mechanisms underlying PTEN expression loss. Emerging evidence suggests that the prognostic significance of *PTEN* loss may vary depending on the type of genomic alteration involved. Combining comprehensive genetic profiling with the current immunohistochemical approach could also uncover more nuanced relationships between PTEN status and tumor immune microenvironment. Additionally, the study population mostly included Caucasians, and the generalizability to other populations needs to be confirmed. Another limitation is that our molecular stratification for CRC was restricted to MMR status, *BRAF* V600E mutation status, and TP53 expression status, as data on other molecular features, such as consensus molecular subtypes (CMS) were not available. Despite the limitations, the large sample size, the availability of extensive clinicopathologic data, and the use of multiplex immunohistochemistry for detailed immune cell phenotyping represent important strengths.

Future studies could also leverage some of the emerging technologies that could help overcome limitations with conventional PTEN immunohistochemistry. For example, AI-assisted image analysis can improve the reproducibility and precision of exact PTEN expression scoring and provide automated quantification of staining intensity and distribution across the entire sample. Additionally, molecular analysis such as fluorescence in situ hybridization (FISH) can differentiate between heterozygous and homozygous *PTEN* deletions that may have distinct prognostic significance. Furthermore, single cell and spatial genomic profiling could provide insights into the mechanisms of PTEN downregulation and enable detailed evaluation of intratumor heterogeneity of PTEN signaling.

Taken together, the findings indicate that immunohistochemical evaluation of PTEN expression alone may not provide clinically meaningful prognostic information in CRC, in contrast to the conclusions of several prior smaller-scale studies. Further research is needed to investigate the potential associations of genomic *PTEN* alterations and tumor immune microenvironment.

## Supplementary Information

Below is the link to the electronic supplementary material.

ESM 1 Supplementary Material 1 (PDF 323 KB)

## Data Availability

Data generated and/or analyzed during this study are not publicly available. The sharing of data will require approval from relevant ethics committees and/or biobanks. Further information including the procedures to obtain and access data of Finnish Biobanks are described at https://finbb.fi/en/fingenious-service.

## Abbreviations

CRC: Colorectal cancer
PTEN: Phosphatase and tensin homolog
PI3K: Phosphoinositide 3-kinase
AKT: Protein kinase B
TME: Tumor microenvironment
MMR: Mismatch repair
