# Supplementary material for: PTEN status, tumor immune microenvironment, and survival in colorectal cancer
Source: Virchows Arch. 2025 Nov 18;489(2):467–76. doi: 10.1007/s00428-025-04327-8 (PMC13477460; doi:10.1007/s00428-025-04327-8)
Supplement: Supplementary file 1 [file 428_2025_4327_MOESM1_ESM.pdf]

Table S1. Antibodies included in the multiplex immunohistochemistry assays.

Macrophage panel
1. CD237 [E1L3N], #13684S, Cell Signaling, 1:100
2. CD3 [LN10], #PA055, Leica, ER1 60min, 1:1
3. PDCD1 [SP269], #ab2277681, Abcam, ER1 30min, 1:40
4. CD163 [10D6], #MS-1103, Thermo Scientific, ER1 60min, 1:100
5. CD86 [E2G8P], #91882S, Cell Signaling, ER1 30min, 1:50
6. HLA-DR [TAL1B5], #sc-53319, Santa Cruz, ER1 30min, 1:4000
7. MRC1 [E2L9N], #91992S, Cell Signaling, ER1 30min, 1:500
8. CD68 [KP1], #916104, BioLegend, ER1 30min, 1:40 000
9. KRT [BS5], #BSH-7124-1, BioSite Histo, ER1 60min, 1:400

Myeloid cell panel
1. CEACAM8 [G10F5], #305102, BioLegend, 1:100
2. FCGR3 [D1N9L], #24316S, Cell Signaling, ER1 30min, 1:70
3. CD14 [D7A2T], #75181S, Cell Signaling, ER1 60min, 1:70
4. HLA-DR [TAL1B5], #sc-53319, Santa Cruz, ER1 60min, 1:4000
5. ITGAM [D6X1N], #49420S, Cell Signaling, ER1 30min, 1:60
6. CD33 [SP266], #ab199432, Abcam, ER1 60min, 1:500
7. TPSAB1 [AA1], #sc-59587, Santa Cruz, ER1 60min, 1:500
8. ARG1 [EPR6672(B)], #ab133543, Abcam, ER1 30min, 1:500
9. IDO [EPR20374], #ab211017, Abcam, ER1 60min, 1:500
10. KRT [BS5], #BSH-7124-1, BioSite Histo, ER1 60min, 1:400

B cell panel
1. CD20 [L26], #Mob004, Diagnostic Biosystems, 1:100
2. CD79A [SP18], #RM-9118, Thermo Scientific, ER1 60min, 1:600
3. IRF4 [MUM1p], #M7259, Dako, ER1 60min, 1:200
4. IgG1 [RM117], #31-1019-00, RevMAb, ER1 60min, 1:4000
5. HLA-DR [TAL1B5], #sc-53319, Santa Cruz, ER1 60min, 1:4000
6. IgG2 [EPR4418], #ab134050, Abcam, ER1 60min, 1:40 000
7. IgG4 [HP6025], #605-900, Thermo Scientificm ER1 60min, 1:500
8. KRT [BS5], #BSH-7124-1, BioSite Histo, ER1 60min, 1:400

Table S2. Multivariable Cox regression models of cancer-specific survival and overall survival for PTEN loss with all variables.

	Cohort 1		Cohort 2	
	Cancer-specific survival	Overall survival	Cancer-specific survival	Overall survival
	Multivariable HR (95% CI)	Multivariable HR (95% CI)	Multivariable HR (95% CI)	Multivariable HR (95% CI)
PTEN status				
Intact	1 (referent)	1 (referent)	1 (referent)	1 (referent)
Reduced	0.83 (0.61–1.15)	0.95 (0.76–1.19	1.06 (0.69–1.64)	1.09 (0.79–1.51)
Lost	1.19 (0.88–1.61)	0.97 (0.76–1.24)	0.85 (0.55–1.31)	0.80 (0.57–1.13)
Age				
<65	1 (referent)	1 (referent)	1 (referent)	1 (referent)
65–75	1.19 (0.91–1.56)	1.39 (1.12–1.73)	1.67 (1.18–2.38)	1.70 (1.26–2.30)
>75	2.03 (1.55–2.65)	3.17 (2.58–3.90)	2.54 (1.74–2.38)	4.11 (3.06–5.52)
Gender				
Male	1 (referent)	1 (referent)	1 (referent)	1 (referent)
Female	0.89 (0.72–1.11)	0.79 (0.67–0.93)	0.98 (0.73–1.31)	0.81 (0.65–1.02)
Year of operation				
2000–2005	1 (referent)	1 (referent)		
2006–2010	0.63 (0.48–0.81)	0.73 (0.67–0.93)	1 (referent)	1 (referent)
2010–2015	0.50 (0.38–0.65)	0.65 (0.53–0.79)	0.79 (0.54–1.16)	0.75 (0.56–0.99)
2016–2020			0.53 (0.36–0.79)	0.62 (0.46–0.83)
Tumor location				
Proximal colon	1 (referent)	1 (referent)	1 (referent)	1 (referent)
Distal colon	0.92 (0.71–1.20)	0.96 (0.78–1.17)	1.09 (0.72–1.67)	1.02 (0.73–1.41)
Rectum	0.80 (0.55–1.16)	0.88 (0.67–1.16)	0.91 (0.60–1.39)	0.97 (0.71–1.34)
Preoperative radiotherapy or chemoradiotherapy				
No	1 (referent)	1 (referent)	1 (referent)	1 (referent)
Yes	1.18 (0.78–1.77)	1.22 (0.90–1.64)	1.30 (0.87–1.94)	1.10 (0.80–1.53)
Disease stage				
I–II	1 (referent)	1 (referent)	1 (referent)	1 (referent)
III	2.96 (2.23–3.92)	1.54 (1.28–1.85)	2.73 (1.76–4.23)	1.46 (1.10–1.93)
IV	18.9 (14.0–25.7)	8.57 (6.84–10.7)	16.2 (10.1–25.9)	7.40 (5.29–10.3)
Tumor grade				
Low-grade (well to moderately differentiated)	1 (referent)	1 (referent)	1 (referent)	1 (referent)
High-grade (poorly differentiated)	1.82 (1.39–2.40)	1.80 (1.46–2.23)	1.53 (1.06–2.19)	1.21 (0.89–1.64)
Lymphovascular invasion				
No	1 (referent)	1 (referent)	1 (referent)	1 (referent)
Yes	1.96 (1.56–2.45)	1.54 (1.28–1.84)	2.17 (1.48–3.17)	1.53 (1.18–1.99)
MMR status				
MMR proficient	1 (referent)	1 (referent)	1 (referent)	1 (referent)
MMR deficient	0.49 (0.30–0.81)	0.65 (0.46–0.90)	0.47 (0.23–0.95)	0.94 (0.62–1.42)
BRAF mutation				
Wild-type	1 (referent)	1 (referent)	1 (referent)	1 (referent)
Mutant	1.36 (0.91–2.03)	1.44 (1.07–1.94)	1.43 (0.82–2.46)	1.36 (0.91–2.03)

Abbreviations: CI, confidence interval; HR, hazard ratio; MMR, mismatch repair.

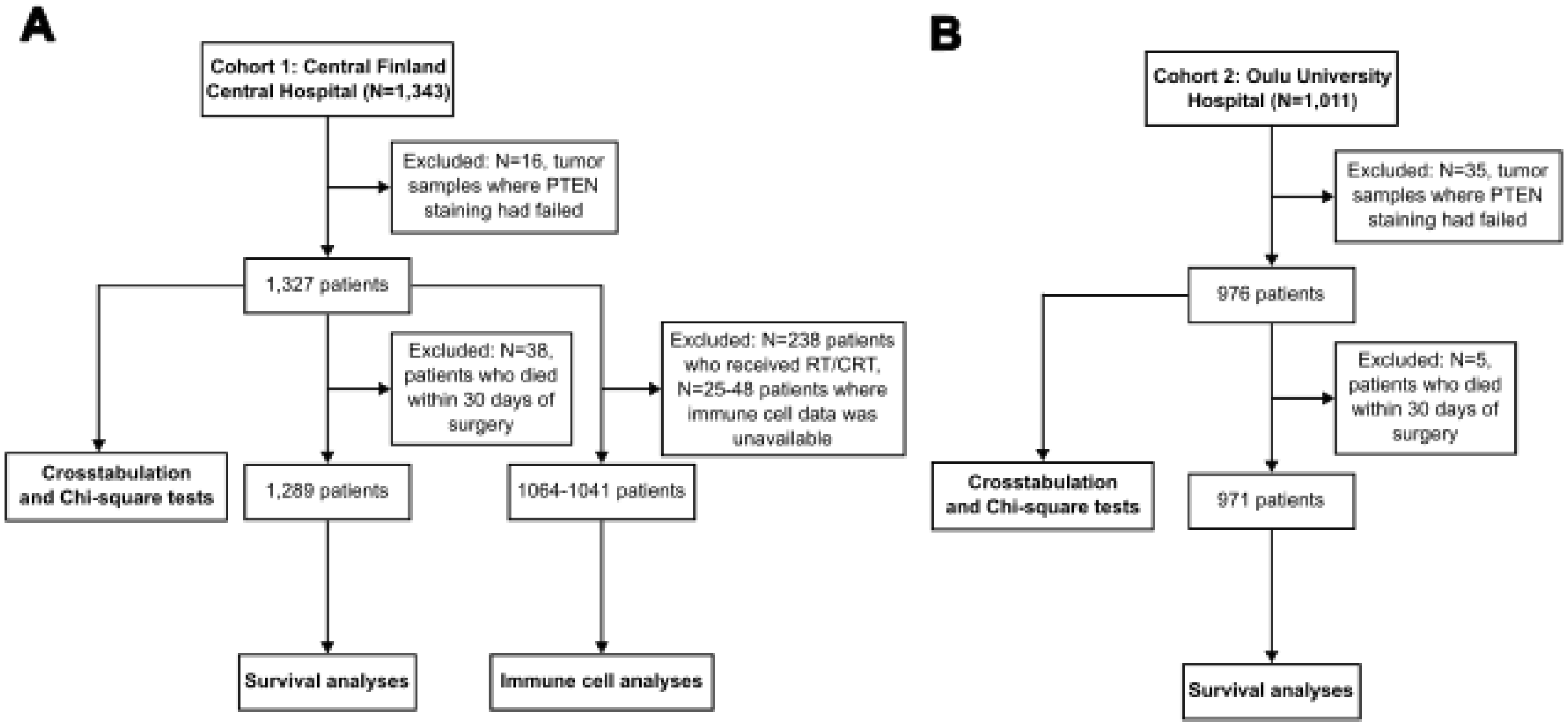

Figure S1. Flowcharts describing the patient selection for the two cohorts. (A) Cohort 1 study population, with varying sample sizes available for different analyses. Note that immune cell analysis was only conducted for cohort 1 and the specific sample sizes for each immune cell type analyzed are indicated in **Fig 3**. (B) Similar flowchart for Cohort 2 study population.
Abbreviations: RT, radiotherapy; CRT, chemoradiotherapy.

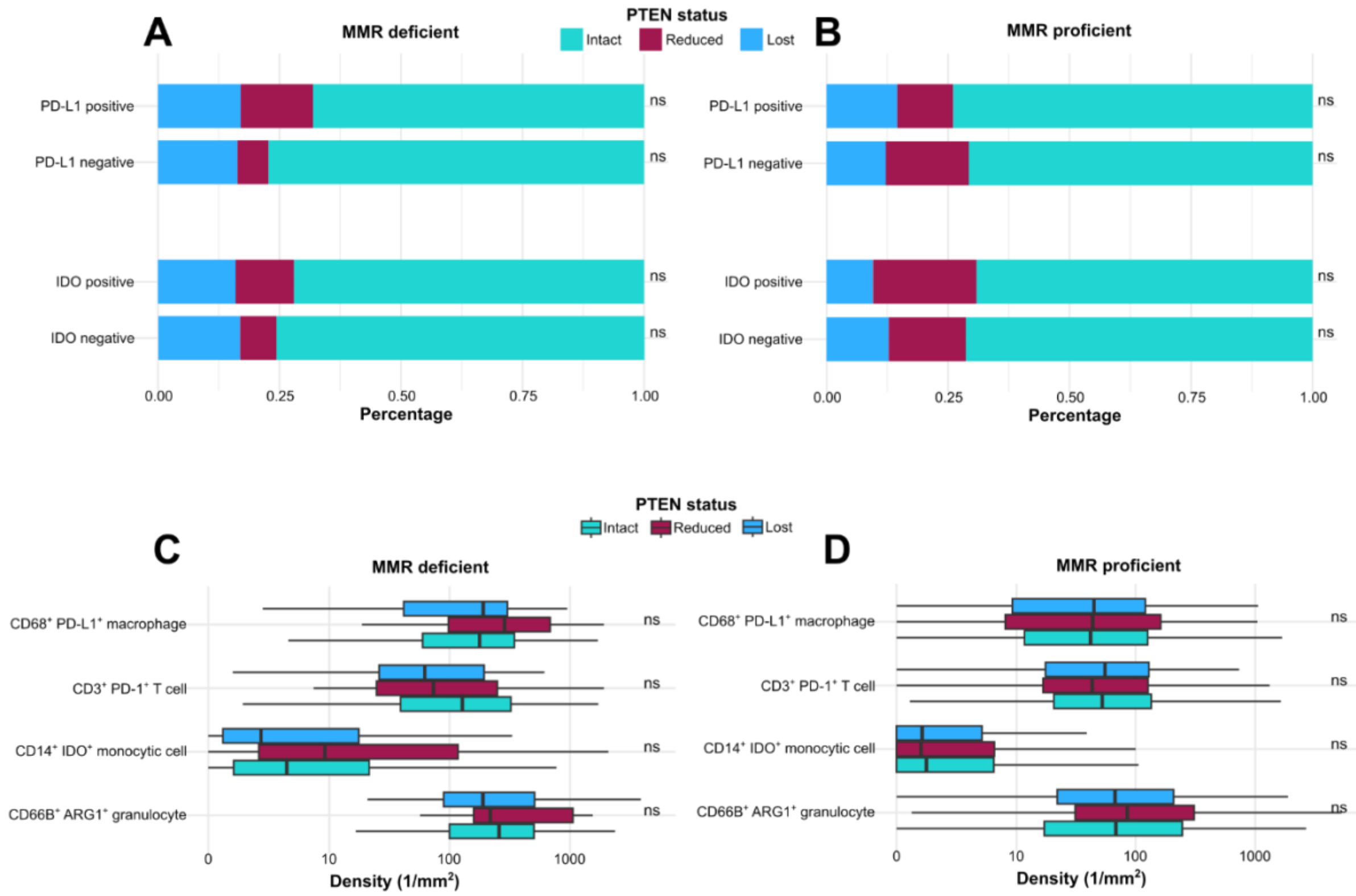

Figure S2. Immunosuppressive factor expression in relation to PTEN status. (A, B) Expression of PD-L1 and IDO in tumor cells across PTEN expression categories. Histoscore positivity cut-off values were 2 for PD-L1 and 5 for IDO. Histoscore evaluation data available; PD-L1 (N=1060); IDO (N=1041). (C, D) $CD68^{+}PD\text{-}L1^{+}$ macrophages and $CD3^{+}PD\text{-}1^{+}$ T cells (N=1060); $CD14^{+}IDO^{+}$ monocytic cells and $CD66^{+}ARG1^{+}$ granulocytes (N=1041). The analysis was stratified by MMR status, considering the effect of MMR status on tumor immune cell composition. PTEN categorization shown at the top, intact expression plotted with green, reduced with red and lost expression with blue. Abbreviations: ns, not significant (Kruskal-Wallis test or Chi-square test p value > 0.05); MMR: Mismatch repair. The analyses were based on Cohort 1.
